# Transcriptional Modulation during Photomorphogenesis in Rice Seedlings

**DOI:** 10.3390/genes15081072

**Published:** 2024-08-14

**Authors:** Parul Gupta, Pankaj Jaiswal

**Affiliations:** Department of Botany and Plant Pathology, Oregon State University, Corvallis, OR 97331, USA; parul.gupta@oregonstate.edu

**Keywords:** rice, photomorphogenesis, RNA-Seq, transcriptome, alternative splicing, lncRNA, MVA and MEP pathways, circadian clock

## Abstract

Light is one of the most important factors regulating plant gene expression patterns, metabolism, physiology, growth, and development. To explore how light may induce or alter transcript splicing, we conducted RNA-Seq-based transcriptome analyses by comparing the samples harvested as etiolated seedlings grown under continuous dark conditions vs. the light-treated green seedlings. The study aims to reveal differentially regulated protein-coding genes and novel long noncoding RNAs (lncRNAs), their light-induced alternative splicing, and their association with biological pathways. We identified 14,766 differentially expressed genes, of which 4369 genes showed alternative splicing. We observed that genes mapped to the plastid-localized methyl-erythritol-phosphate (MEP) pathway were light-upregulated compared to the cytosolic mevalonate (MVA) pathway genes. Many of these genes also undergo splicing. These pathways provide crucial metabolite precursors for the biosynthesis of secondary metabolic compounds needed for chloroplast biogenesis, the establishment of a successful photosynthetic apparatus, and photomorphogenesis. In the chromosome-wide survey of the light-induced transcriptome, we observed intron retention as the most predominant splicing event. In addition, we identified 1709 novel lncRNA transcripts in our transcriptome data. This study provides insights on light-regulated gene expression and alternative splicing in rice.

## 1. Introduction

Light is an essential growth factor for sustaining autotrophic plant life. The quality, quantity, direction, and length of light exposure affect plant growth and development [1,2,3]. The natural light–dark cycle maintains the carbon and nitrogen metabolism of rice plants [4]. Light induces transcriptional reprogramming in various plant species [4,5,6,7,8,9,10,11,12] via an array of photoreceptors [13,14]. The photoreceptors phytochrome (red light) and cryptochrome (blue light) are involved in light signaling and photomorphogenesis [15,16]. Genes encoding phytochrome interacting factors (PIFs) promote skotomorphogenesis and development in the dark [17,18,19]. Light-mediated gene expression modulation is also triggered by translational enhancement of the pre-existing mRNA pool instead of an enhanced transcription rate [20,21].

Intron splicing in multiexonic mRNA is a post-transcriptional regulatory process that often produces different mRNA isoforms transcribed from a single gene locus, thus contributing to proteome plasticity. Light-induced alternative splicing (AS) was observed in ~10% of the protein-coding genes of *Arabidopsis* and *Physcomitrella patens* [22,23]. A flash of light applied in the middle of the dark or nighttime is sufficient to induce splicing [24]. In contrast to animals, where the most common AS event is exon skipping (ES), intron retention (IR) is the most common AS event in rice [25], Arabidopsis [25,26], poplar [27], and *Salvia hispanica* (Chia) [28]. It is now well-established that transcriptome modulation via AS is vital for plant growth, development, and stress response [29,30,31].

Compared to the protein-coding genes, long non-coding RNAs (lncRNAs) are transcripts >200 bp in length that do not have protein-coding potential. lncRNAs are classified as follows: (i) sense lncRNAs, (ii) antisense lncRNAs, (iii) intergenic RNAs (lincRNAs), (iv) intronic RNAs, and (v) bidirectional lncRNAs [32]. In addition to the numerous studies coupling gene expression and AS studies on protein-coding genes, it is now known that lncRNAs also play a role in regulating gene expression through transcriptional, post-transcriptional, and chromatin remodeling mechanisms [32,33,34,35]. In rice, lncRNAs regulate biological processes such as ovule development, female gametophyte abortion [36], sexual reproduction [37], stress response [38,39], and meiosis and low fertility in autotetraploids [40]. However, the role of light in regulating lncRNAs is not well studied. *Arabidopsis* non-coding RNA *HID1* is a known positive regulator of photomorphogenesis in continuous red light [41].

Rice is a global staple crop and is a model for studying crop genomics. The transition from skotomorphogenesis under dark conditions to photomorphogenesis under light exposure is critical for seedling survival and requires precise control of gene expression by different regulatory mechanisms. High-throughput gene expression datasets are valuable sources for the detection of alternative transcripts, novel transcript isoforms, protein-coding and noncoding genes, improved annotation of plant transcriptomes, and studying developmental processes [42,43,44].

## 2. Materials and Methods

### 2.1. Plant Material, Growth Conditions, and Treatment

Seeds of rice (*Oryza sativa* spp. *japonica* cv. Nipponbare) were grown and processed for the experiment by following the growth conditions and sampling described previously [45]. Seeds were sown in the dark and germinated on day 2. After sowing, these germinated seedlings grew in the dark for 8 days. At the end of day 8, three biological replicates of the dark-grown etiolated shoots were harvested. The remaining dark-grown seedlings were exposed to continuous white light at 120 µmol/m^2^/sec (measured at the soil surface) for 48 h or 2 days (day 9 and 10 after sowing). The shoots of three biological replicates of light-treated green-colored seedling samples were harvested at the end of day 10. Harvested samples were frozen using liquid nitrogen and stored at −80 °C until further processing. Data analysis is described for light regulation.

### 2.2. Sample Preparation and Sequencing

Total RNA from frozen tissue samples was extracted using RNA Plant reagent (Thermo Fisher Scientific Inc., Waltham, MA, USA) and RNeasy kits (Qiagen Inc., Germantown, MD, USA) and treated with RNase-free DNase (Thermo Fisher Scientific Inc., USA) according to the manufacturer’s protocol. The total RNA quality and concentration were determined using an ND-1000 spectrophotometer (Thermo Fisher Scientific Inc., USA) and Bioanalyzer 2100 (Agilent Technologies Inc., Madison, WI, USA). PolyA-enriched mRNA libraries were prepared from three biological replicates of dark and light samples using the TruSeqTM RNA Sample Preparation Kits (v2) and sequenced as 51 bp single-end reads using the Illumina HiSeq 2000 instrument (Illumina Inc., San Diego, CA, USA) according to the manufacturer’s protocol at the Center for Genomic Research and Biocomputing, Oregon State University. The strand-specific sequencing reads and metadata were deposited at EMBL-EBI ArrayExpress (accession number E-MTAB-5689).

### 2.3. RNA-seq Data Analysis

The generation of FASTQ files from the RNA-Seq sequences was performed by CASAVA software v1.8.2 (Illumina Inc.). Sequence reads were filtered and trimmed for low quality at a score of 20 using Sickle v1.33 [46]. Clean, high-quality reads from each sample and replicates were aligned to the *Osj* cv Nipponbare reference genome (IRGSP-1.0.31) using TopHat v2.1.1 [47]. Mapped reads were assembled using Cufflinks, and the reference-guided assembled transcripts from each replicate were merged using Cuffmerge [48]. Assembled transcripts were compared to the reference genome annotation using Cuffcompare. The RSEM software package estimated normalized baseline expression from the aligned sequence reads [49]. For differential gene expression analysis, read count data obtained from RSEM were used in EBSeq [50]. Differentially expressed (DE) genes were filtered based on the false discovery rate-corrected *p* value ≤ 0.05.

### 2.4. Functional Annotation

We carried out the Gene Ontology (GO) enrichment analysis tool provided by the GO consortium [51] to determine the biological roles the enriched gene set played. Plant pathway enrichment analysis was performed by mapping the DE genes using the Plant Reactome analysis tool [52,53,54] (http://plantreactome.gramene.org/PathwayBrowser/#TOOL=AT accessed on 16 August 2023).

### 2.5. Alternative Splicing Analysis

The SpliceGrapher v0.2.5 pipeline identified splicing events in the transcripts from the samples [55]. Sequence reads from each sample, and from replicates, were aligned to the reference rice genome. Splice site-specific classifiers were built using build_classifiers.py script using canonical (GT) and noncanonical (GC) donor sites and acceptor site (AG) for *Os* genome annotation version 31 (Oryza_sativa.IRGSP-1.0.31). Read alignments in SAM format from each replicate were used as input for SpliceGrapher’s sam_filter.py script to filter out false-positive sites. SpliceGrapher Python scripts were used for the generation of depth files (sam_to_depths.py), splice graph prediction (predict_graphs.py), generating statistics (splicegraph_statistics.py) from a set of splice graphs, gene-by-gene summary (genewise_statistics.py) of splicing events, and splice graph visualization (plotter.py). The Realignment pipeline was used to construct putative transcripts from unresolved exons with sufficient coverage from the alignments [55]. In the following steps, IsoLasso [56], an extension of the SpliceGrapher workflow, was used to predict novel splicing events.

### 2.6. Prediction of Long Non-Coding RNA (lncRNA)

All transcripts annotated as intergenic transcripts, intron transcripts, antisense exon transcripts overlapping the reference exons, and antisense intron transcripts overlapping the reference introns were considered potential lncRNA candidates. Transcript sequences of length ≤200 nucleotides were filtered out, and the gffread function of Cufflinks was used to extract fasta sequences of potential lncRNA transcripts from the gtf file. CPC2 [57] was used to predict the coding potential of transcripts. Predicted lncRNAs were scanned by InterProScan [58] to ensure the absence of protein-coding domains. To identify novel lncRNAs, a BLASTn [59] search was performed against a custom BLAST database generated using rice lncRNAs downloaded from the CANTATAdb2.0 (http://cantata.amu.edu.pl accessed on 16 August 2023), PNRD [60], GreeNC [61], and RiceLNCPedia [62] databases. Secondary structures for the lncRNA molecules were predicted by the RNAfold software [63,64].

## 3. Results

### 3.1. Light-Mediated Differential Gene Expression during Photomorphogenesis

**Figure 1:**
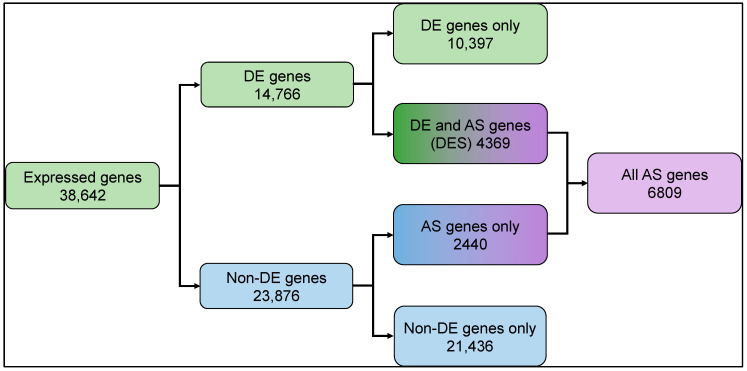
Summary of differential gene expression and transcript splicing observed in rice seedling shoots undergoing photomorphogenesis. Expressed genes: total number of expressed genes in rice transcriptome; DE genes: differentially expressed genes; Non-DE genes: genes with no significant difference in expression; AS: alternatively spliced; DES: differentially expressed and spliced. Expressed and differentially expressed gene counts are represented by green shaded boxes, non-DE genes in blue shaded boxes, and AS genes in purple boxes. Mixed color boxes represent gene counts from overlapping AS, DE, and non-DE.

To explore the transcriptome modulation in rice in response to light, we sequenced the strand-specific poly-A enriched RNA fraction isolated from three biological replicates of rice plants grown under dark and light exposure conditions (see methods). A total of ~38 million and ~42 million high-quality reads were generated from the dark- and light-treated samples, respectively. More than 92% of reads from each sample aligned to the rice reference genome (Supplementary Table S1). We found 38,642 genes showed baseline normalized expression in the samples, of which 33,943 genes expressed in the dark vs. 35,772 genes that expressed under light, respectively. Differential expression analysis identified 14,766 light-regulated genes (Figure 1, Supplementary Figure S1, Supplementary Table S2).

### 3.2. Gene Function and Pathway Enrichment Analyses

Gene Ontology-based functional annotation analysis of the light-regulated differentially expressed gene set (Supplementary Figure S2) revealed enrichment for biological processes (BP), such as chlorophyll and cell wall biosynthesis, besides photosystem light reaction pathways and ribosome assembly. Dominant molecular functions (MF) were rRNA binding, cytoskeletal motor activity, oxidoreductase and metallopeptidase activities, translation elongation, and chaperone binding. As expected, chloroplasts, cytoskeleton, ribosomes, peroxisomes, and nucleus were the major cellular component sites of activity.

**Figure 2:**
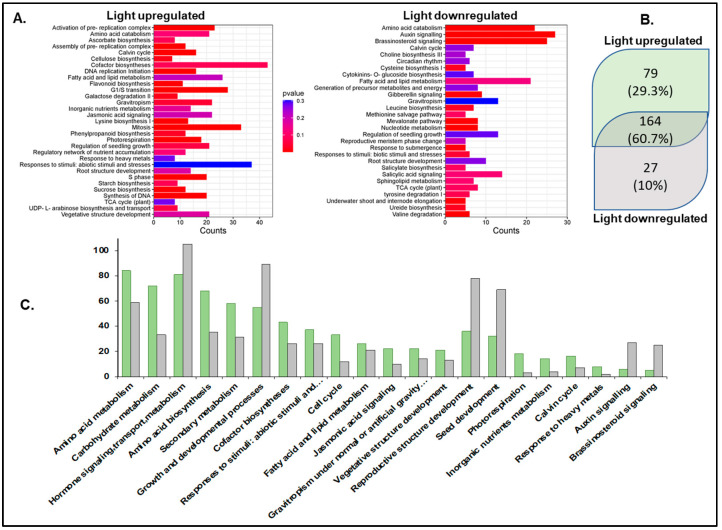
Pathway enrichment analysis using the Plant Reactome. (**A**) Plant Reactome pathway enrichment analysis plots. (**B**) Unique and shared pathways enriched for the light-upregulated and downregulated gene sets. (**C**) Counts of genes mapped to some of the common Plant Reactome pathways. Light-upregulated (green) and downregulated (grey).

Pathway enrichment analysis using the Plant Reactome pathway analysis tool [65,66] mapped 475 light-upregulated genes to 243 pathways (Supplementary Table S3). A total of 164 pathways overlapped between light-up- and light-downregulated genes. Most pathways showed higher mappings to light-upregulated genes, except hormone auxin and brassinosteroid signaling and reproductive structure development (seed). Pathways unique to light-upregulated genes include those for the biosynthesis of photosynthesis components such as chlorophyll, carotenoid, and phylloquinone, hormones like gibberellin, auxin, abscisic acid, etc. The pathways with unique mapping to light-downregulated genes include polar auxin transport, mevalonate (MVA) pathway, circadian clock, salicylic acid metabolism and signaling, reproductive plant part development, and root-specific gene network of NAC10 transcription factor (Figure 2).

### 3.3. Identifying Light-Regulated Transcription Factors

To identify the light-regulated transcription factors (TF), a list of rice TFs was downloaded from the Plant Transcription Factor Database [67] and searched against the DE genes. We found 429 light-upregulated and 498 light-downregulated TFs (Supplementary Table S4). We found *WRKY*, *NAC*, and orphans were the most abundantly expressed TFs in light, compared to many light-downregulated *bHLH*, *bZIP*, and *C3H* gene family members (Table 1). Highly upregulated (fold change ≥10) TFs belong to the *MYB*, *AP2-EREBP*, *WRKY*, *Orphans*, *NAC, MADS*, and *bHLH* gene families; however, downregulated TFs belong to the *AP2-EREBP, C2C2-CO-like*, *C3H*, *HB*, and *NAC* gene families (Supplementary Table S4). To investigate whether TFs targeted the MVA and MEP pathway genes, we surveyed the list of TFs and their potential targets identified by the Plant Transcription Factor Database. We identified 23 TFs that potentially bind to the promoter region of six MVA pathway genes. Two MVA pathway genes, hydroxymethylglutaryl-CoA synthase (*HMGS*; Os08g0544900) and 3-hydroxy-3-methylglutaryl coenzyme A reductase (*HMGR*; Os08g0512700), were targeted by bZIP and AP2-EREBP factors, respectively, whereas the mevalonate 5-diphosphate decarboxylase (*MDD*; Os02g0109100) gene was a target of 13 AP2-EREBPs, one C3H protein, and one C2H2 protein (Supplementary Table S5). None of the TFs we found bind to the promoter of MEP pathway genes.

### 3.4. Transcript Splicing during Photomorphogenesis

To study how light exposure modulates transcript splicing (AS) events in the rice seedling shoots, we analyzed the transcriptome data by using SpliceGrapher v0.2.5 [55]. We created highly accurate splice site classifiers for rice with canonical (GT-AG) and semi-canonical (GC-AG) splice sites to filter the splice junctions in the sequence reads aligned to the reference genome. After removing the false positive splice sites, we predicted chromosome-wise splice graphs from the two transcriptome sample datasets. In the dark-treated dataset, 63.9% were true positive junctions, with 5.5% novel splice sites. In the light-treated dataset, 64.3% of splice junctions were true positives, of which 5.8% were predicted as novel sites. We observed 6214 spliced genes with 9685 splicing events in the dark samples compared to the 6809 spliced genes with 10,432 splicing events in the light samples (Figure 1, Table 2). The highest number of spliced genes and events was on chromosome 1, and the lowest was on chromosome 10 (Table 2). Intron retention (IR) was the most prevalent type of splicing event in both samples, followed by exon skipping (ES). Alternative 3′ splicing (Alt.3′) was the least common event. We realigned the sequenced reads from the two samples to the putative transcripts to resolve ambiguous donor and acceptor splice site combinations. We resolved the novel exons to generate splice graphs. Light induced splicing in 2162 unique genes compared to 1567 unique genes that undergo splicing in the dark. Of the 4647 spliced genes shared between the two samples, 165 displayed differential splicing events.

In our differentially expressed and spliced genes (DES), we found that approximately 30% (4369) differentially expressed (DE) genes also undergo splicing (AS). Approximately 10% (2440) of the non-DE genes undergo splicing (Figure 1). Light-upregulated DES genes show enrichment for various molecular functions like oxidoreductase, hydrolase, isomerase activity, and RNA binding, which play roles in photosynthesis, transmembrane transport, and small molecule metabolic process, and are located in the cellular components chloroplasts and membrane-bound organelles. In contrast, the light-downregulated DES genes are enriched for RNA processing, response to stress, protein binding, and macromolecule metabolic process, and are localized in the cytoplasm and nucleus cellular components (Supplementary Figure S3).

We also surveyed the DES aspects of the 57 known spliced genes in rice [25,68] and observed splicing in 41 genes (Table 3). Of these, seven spliced under dark conditions, three under light conditions, and thirty-one genes spliced under both dark and light conditions. Of the known genes, we also observed that 18 genes were light-upregulated and 24 downregulated. Only 32 genes showed a DES profile. Three light-upregulated genes (Os02g0130600, Os05g0348100, Os12g0567300) show complete splicing in light compared to intron retention in the dark. In contrast, the two light-downregulated genes (Os02g0666200 and Os04g0656100) show intron retention.

### 3.5. Light-Mediated Shift in Gene Expression and Splicing Events

#### 3.5.1. Circadian Clock Pathway Genes

The circadian clock is entrained by light and the diurnal photoperiod-regulated clock genes [69,70]. We investigated the light-regulated DES profile of clock genes. Most of the circadian clock genes spliced under either or both conditions. Four genes, *Casein kinase alpha subunit* (*CK2α-1/Hd6*, Os03g0762000), *Casein kinase beta subunit* (*CK2β-2*, Os10g0564900), *Phytochrome B* (*PhyB*, Os03g0309200), and *Timing of CAB Expression 1* (*TOC1*, Os02g0618200), showed differential splicing (only reference splicing events or complete splicing and no novel isoforms) upon light treatment compared to the presence of an unspliced intron in the absence of light (Supplementary Figure S4). In contrast, only one gene, *Phytochrome Interacting Factor 3* (*PIF3*, Os01g0286100), showed intron retention under light. All five genes were upregulated in light (Supplementary Figure S4). Seven genes, *Casein kinase alpha subunit* (*CK2α-2*, Os03g0763000), *Casein kinase beta subunit* (*CK2β-1*, Os07g0495100), blue light receptor *Cryptochrome 2* (*CRY2*, Os02g0625000), *Gigantea* (*GI*, Os01g0182600), *Pseudo-Response Regulator 37* or *Heading date 2* (*PRR37*, *Hd2*, Os07g0695100), and *Pseudo-Response Regulator 73* (*PRR73*, Os03g0284100), showed differential splicing under both conditions (Supplementary Figures S4 and S5). Exon skipping and alternative 5′ (Alt.5′) splicing events in the *CK2α-2* (Os03g0763000) gene were induced in light. Both *CK2β-1* (Os07g0495100) and *GI* (Os01g0182600) displayed incomplete splicing of intron (IR) in the dark. *PRR37* showed three types of splicing events (Alt.3′, ES, and IR) under light, whereas the second IR event observed toward the 3’ end under dark was spliced in light. Conversely, *PRR73* showed three types of events (Alt.3′, ES, and IR) under dark conditions, whereas under light conditions, two new Alt.5′ events were identified. Expression of both PRR genes (*PRR37* and *PRR73*) was light-downregulated (Supplementary Figure S4). Light-upregulated *PRR95* shows an additional unspliced intron in light.

#### 3.5.2. MVA and MEP Pathway Genes

**Figure 3:**
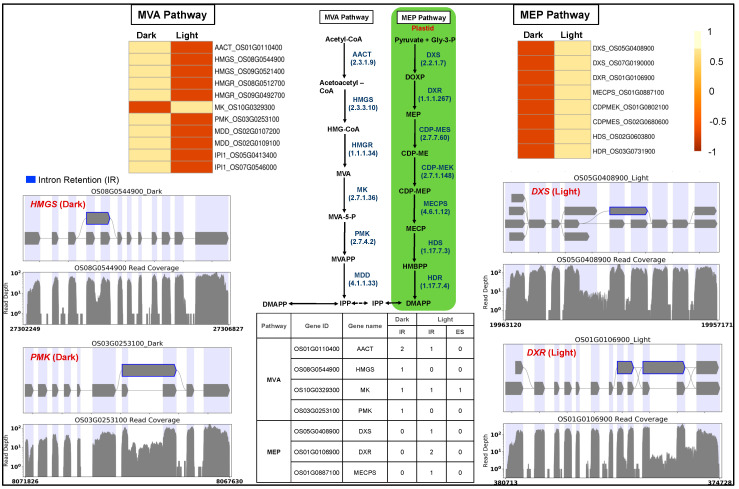
Gene expression and splicing of cytosolic mevalonate (MVA) and plastid-localized methyl erythritol 4-phosphate (MEP) pathway genes. *AACT*, acetyl-CoA acetyltransferase; *HMGS*, hydroxymethyl glutaryl CoA synthase; *HMGR*, 3-hydroxy-3-methylglutaryl-coenzyme A reductase; *MK*, mevalonate kinase; *PMK*, phosphomevalonate kinase; *MDD*, mevalonate diphosphate decarboxylase; *DXS*, 1-deoxy-D-xylulose-5-phosphate synthase; *DXR*, 1-deoxy-D-xylulose-5-phosphate reductoisomerase; *CDPMES*, 2-C-methyl-D-erythritol4-phosphate cytidylyl transferase; *CDPMEK*, 4-diphosphocytidyl-2-C-methyl-D-erythritol kinase; *MECPS*, 2-C-methyl-D-erythritol2,4-cyclodiphosphate synthase; *HDS*, 4-hydroxy-3-methylbut-2-enyldiphosphate synthase; *HDR*, 4-hydroxy-3-methylbut-2-enyldiphosphate reductase. Blue: intron retention.

Light regulates the biosynthesis and accumulation of secondary plant products like isoprenoid-derived metabolites required for plant growth and development. Isoprenoid precursors are synthesized via cytosolic MVA and plastid-localized MEP pathways, their fluxes are regulated by light [71,72], and their products are essential for the successful development and function of chloroplast. We observed that MEP pathway genes were light-upregulated compared to the downregulated MVA pathway genes (Figure 3). Light positively regulates MEP pathway activity, whereas MVA pathway activity appears negatively regulated by light [73].

We investigated the light-induced differential splicing events in the MEP and MVA pathway genes. Four MVA pathway genes (acetyl-CoA acetyltransferase: AACT, hydroxymethyl glutaryl CoA synthase: HMGS, mevalonate kinase: MK, phosphomevalonate kinase: PMK) and three MEP pathway genes (1-deoxy-D-xylulose-5-phosphate synthase: DXS, 1-deoxy-D-xylulose-5-phosphate reductoisomerase: DXR, 2-C-methyl-D-erythritol2,4-cyclodiphosphate synthase: MECPS) showed intron retention events in the dark (Figure 3). The gene MK showed a novel light-induced exon-skipping event.

### 3.6. lncRNA Discovery and Splicing during Photomorphogenesis

**Figure 4:**
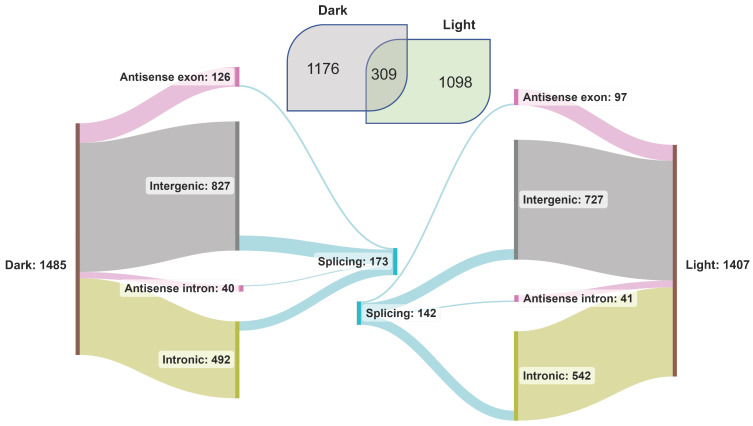
Classification of lncRNAs identified in the transcriptome of dark- and light-treated rice seedling samples.

lncRNAs are known to regulate diverse biological processes in plants, including photomorphogenesis [74], and as described above, are categorized into four major types based on their location in the gene space and on the DNA strand. We used our transcriptome data to characterize light-induced rice lncRNAs (Figure 4). We identified 1485 and 1407 lncRNA transcripts in the dark and light samples, of which 309 were common. Many lncRNAs from intergenic and intronic regions were identified (Figure 4). To discover novel lncRNAs, the transcripts from our datasets were queried against the custom BLAST database of rice lncRNAs. About 44% of the total lncRNA transcripts (2583) found a match in the BLAST database (Supplementary Table S6). A total of 173 lncRNAs spliced under dark and 142 spliced under light conditions, most located in the intergenic regions (Figure 4). Interestingly, all the spliced lncRNA genes from both datasets were from Chr1, Chr10, Chr11, and Chr12. The maximum number of spliced lncRNA genes was from Chr1, followed by Chr11, 12, and 10. Among all the splicing events, IR was predominant under both conditions.

We found lncRNA transcripts from light (TCONS_00012152) and dark (TCONS_00011794) datasets that were present on the antisense strand overlapping an exon region of the *MADS27* (Os02g0579600) gene. The transcript from the light-treated sample appears longer and showed two alternative 3’ splice sites and one exon skipping event. The TCONS_00011794 may compete with *MADS27* to form a complex with miRNA osa-miR444a known to play multiple roles in the nitrate-dependent development pathway [75]. Among the list of lncRNAs present on the antisense strand overlapping annotated genes, we identified a light-induced lncRNA (TCONS_00001515) overlapping *ERF99* transcription factor (Os01g0868000) exon that may play a role in its silencing. *ERF99* plays a central role in mediating abiotic stress responses [76]. Similarly, a lncRNA transcript (TCONS_00015825) is present exclusively in light and is transcribed from the intronic region of the antisense strand of a B-type response regulator gene (Os03g0224200), known for its involvement in cytokinin signaling, meristem maintenance, and stress response [77,78]. The lncRNA transcript TCONS_00026617, present exclusively in the dark, overlaps the intronic region of a phytochrome-interacting factor gene *PIF14* (Os07g0143200). *PIF14* is known to bind the active form of phytochrome B and plays a crucial role in cross-talk between light and stress signaling [79].

## 4. Discussion

Light is known to induce changes in the transcriptome, metabolome, and proteome of plants [80], and thereby not only regulates the development, function, and physiology of the chloroplast but also provides signals for modulating the plant’s morphological, developmental, and physiological adaptions in response to growth environments [7,45,81,82,83]. Though plants experience constantly changing light conditions under the natural environment daily during their lifetime, early development from germination to the seedling stage and acquiring the full autotrophic capability is very much programmed by exposure to light. Therefore, we investigated transcriptome modulation in rice seedlings during photomorphogenesis. Compared to earlier studies in rice and *Arabidopsis*, where ~20% of the genes were reported to be differentially expressed in dark-grown etiolated seedlings compared to light-exposed green seedlings [6,8,84], we observed that the transition from skotomorphogenesis to photomorphogenesis alters the differential expression of ~38% of the rice genes.

Functional annotation of the differentially expressed genes showed enrichment for roles in secondary metabolism, chloroplast-related biosynthetic pathways, hormone biosynthesis and signaling, amino acid biosynthesis, fatty acid metabolism, etc. (Figure 2, Supplementary Table S3). This result was expected based on the earlier reports on light regulation of such events [24,85].

**Figure 5:**
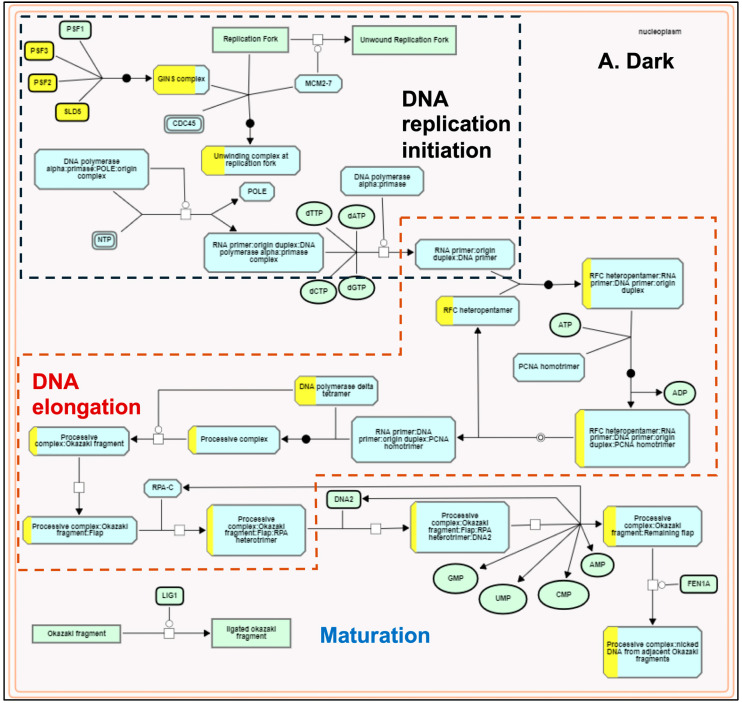

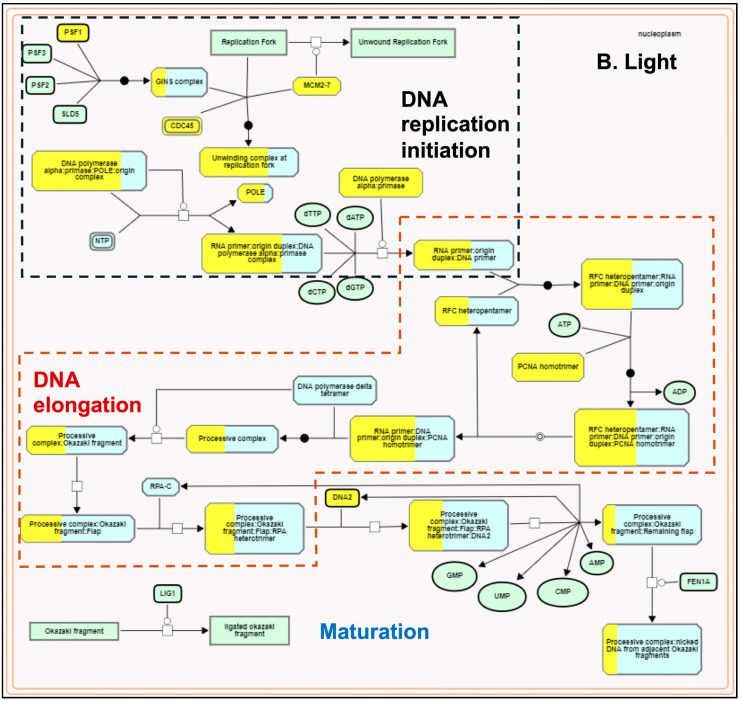
Plant Reactome pathway enrichment showing light-regulated genes and reaction event coverage for the DNA biosynthesis pathway of the G1-to-S phase mitotic cell cycle. (**A**) shows enrichment in dark conditions and (**B**) shows enrichment in light conditions. Each reaction has several input and output entities and may be regulated or catalyzed by a protein and or a protein complex. Solid arrows depict the directionality. The view also shows overlaps between the sub-pathways. The boxes with yellow-painted areas show the gene coverage (the number of genes mapped to the protein or protein complex entity participating in the respective reaction).

The plastids develop into chloroplasts by following an essential step of developing the thylakoid membrane system and recruiting and assembling components of light and dark reactions to establish a functional photosynthetic process. Many terpenoid compounds are essential to plants’ light-harvesting function and protect against reactive oxygen species (ROS) damage. The basic isoprenoid units for terpenoid biosynthesis, such as tocopherols, plastoquinones, carotenoids, chlorophylls, and precursors of the growth hormones gibberellins and abscisic acid, are synthesized by the plastid-localized MEP pathway [86,87]. At the same time, isoprenoid units synthesized by the MVA pathway contribute to the synthesis of triterpenes, phytosterols, and phytohormones [88]. MEP and MVA pathways complement and contribute to the biosynthesis of chlorophylls and carotenoids required for plastid development [89]. MEP pathway genes were light-upregulated in our transcriptome data compared to the light-downregulated MVA pathway genes (Figure 2). These results are consistent with earlier reports [90,91] and confirm that light acts as a critical regulator in modulating the availability of isoprenoid precursors during photomorphogenesis [92]. Arabidopsis circadian clock genes (*LHY*, *PRR9*, *CCA1*, *TOC1*) regulate MVA and MEP pathways [73]. *AtTOC1* is also known to regulate the G1-to-S phase transition in the mitotic cell cycle in early leaf development [93]. In our dataset, we did not observe any significant change in the expression of *LHY/CCA1* (Os08g0157600), *PRR95* (Os09g0532400, a homolog of *AtPRR9*), and *TOC1* (Os02g0618200). However, all three genes spliced differently. Under the light, *TOC1* showed no splicing, but it retained two introns in the dark. We observed one exon-skipping event of the *LHY/CCA1* gene in light, whereas *PRR95* showed one additional intron retention (Supplementary Figure S4). Similar to *Arabidopsis* [93], the functional enrichment analysis also identified the *TOC1*-regulated DNA biosynthesis pathway of the G1-to-S transition of the mitotic cell cycle (Figure 5) along with a greater coverage of reaction events in the light-upregulated set. To our knowledge, this is the first report on the light regulation of the mitotic cell cycle pathway in rice.

Previous transcriptome studies showed that ~42% of *Arabidopsis*, ~63% of *soybean*, and ~56% of maize genes undergo alternative splicing events [92,94,95]. Studies on rice by Wang and Brendel [25] reported IR for 69% of 6568 AS genes, and Campbell et al. [96] reported 44.7% of 8772 AS genes. In our dataset, 4369 genes undergo differential expression and alternative splicing, called DES genes (Figure 1A). About 80% (5395) of the spliced genes (6809) show IR events, suggesting that IR is the major splicing event. The more significant number of splicing events in the light-treated samples suggests that light-mediated gene expression and post-transcriptional mRNA splicing play an important role in photomorphogenesis. The splicing events in rice circadian clock genes suggest that IR regulates the expression of PRR genes (Supplementary Figure S4). Many of the light-regulated genes of the MEP and MVA pathways show IR events, suggesting that light plays a positive role in completing the splicing and provides a hypothesis that exposure to light is an adaptive feature and, even if the gene is expressed in dark, it stays unspliced until it encounters light. Our data show the presence of all three states, partially spliced in the dark (*GI*, Os01g0182600), fully spliced in light (*GI*, Os01g0182600), and both states in light (*CK2β-1*, Os07g0495100) (Supplementary Figure S5).

lncRNAs play regulatory functions in essential biological processes such as vernalization, photomorphogenesis, and stress regulation [27,41,97], and display splicing [98]. A total of 6480 long intergenic non-coding RNAs (lincRNAs) were identified from 200 *Arabidopsis* transcriptome datasets [99]; however, we identified 827 lincRNAs in the dark and 727 lincRNAs in the light datasets. Corona-Gomez and coworkers [100] characterized the splicing conservation of lncRNAs in *Brassicaceae*, which revealed that ~18% of lincRNAs display splicing conservation in at least one exon. In our dataset, ~10% of lncRNA transcripts undergo splicing, and the majority are of long intergenic non-coding RNA type (Figure 4). Komiya and colleagues reported [101] that phased small interfering RNAs (phasiRNAs) are generated from over 700 lincRNAs, and these phasiRNAs bind to rice argonaute protein MEL1. MEL1 has a specific function in developing pre-meiotic germ cells and the progression of meiosis. In our dataset, many lincRNAs transcribed from MEL1-phasiRNA clusters are apparent.

**Figure 6:**
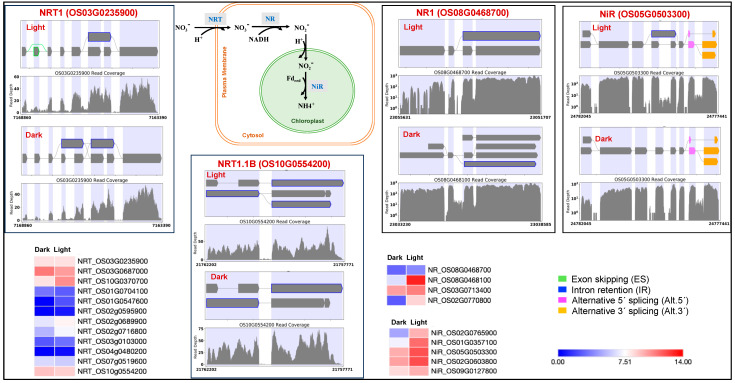
Splicing and expression pattern (normalized counts) of nitrogen assimilation cycle genes under dark and light conditions. NRT: nitrate transporter; NR: nitrate reductase; NiR: nitrite reductase.

Identification of light-induced lncRNAs antisense to *ERF99*, B-type response regulator genic region may play a role in transcriptional regulation. *ERF99* is known to modulate root architecture and to be downregulated in crown root primordia [102]. The *MADS27* gene is targeted by lncRNAs present in both dark and light conditions. However, the dark lncRNA (TCONS_00011794) is longer and, based on its predicted RNA fold structure and lower free energy, is likely more stable than its transcript TCONS_00012152 in light. For the MADS27 gene, we also observed an additional IR event in light. The MADS27-miR444 complex is known to play a role in plant development in a nitrate-dependent manner [103,104,105]. Therefore, we investigated the nitrogen assimilation pathway genes. Nitrogen assimilation is necessary for sustaining plant growth and development. Various nitrogen assimilatory enzymes are known to show isoform and cellular component-specific responses under light and dark conditions [106,107]. We observed light upregulation for the nitrate transporter, nitrate reductase, and nitrite reductase genes. We also observed differential splicing patterns for these genes (Figure 6). We also found light regulation of the recently reported rice abiotic and biotic stress response genes [108,109]. We expect this study’s condition-dependent novel splice events will help improve the annotation of the rice reference genome and pangenome [54,110]. 

## 5. Conclusions

This study suggests that light is a significant regulatory factor controlling genome-wide gene expression through an alternative splicing mechanism in rice. All spliced genes did not necessarily produce novel isoforms, which indicates the coupling of AS and nonsense-mediated decay (NMD). NMD prevents the translation of mutant mRNAs harboring potential premature stop codons by targeting them for degradation. In *Arabidopsis*, 77.2% of light-regulated AS events exhibit NMD features within a splicing isoform [111]. We conclude that light induces a significant number of splicing events in rice protein-coding and non-coding lncRNA genes. This photomorphogenesis transcriptome study is a valuable resource for lncRNA research in rice and provides insights into the portion of the genome regulated at the level of alternative splicing in response to light.

## Figures and Tables

**Table 1:** Transcription factor gene families and the number of members showing differential expression in response to light.

Members of light-regulated transcription factor gene families					
Gene family	Upregulated	Downregulated	Gene family	Upregulated	Downregulated
ABI3VP1	6	4	GRF	1	2
Alfin-like	1	4	HB	21	24
AP2-EREBP	23	32	HSF	3	10
ARF	8	8	LIM	2	1
ARR-B	1	4	LOB	2	1
BBR-BPC	0	1	MADS	6	5
BES1	0	3	mTERF	9	4
bHLH	19	27	MYB	28	14
BSD	1	6	MYB-related	16	25
bZIP	10	27	NAC	21	19
C2C2-CO-like	4	7	OFP	2	1
C2C2-Dof	5	4	Orphans	20	10
C2C2-GATA	7	4	PBF-2-like	1	0
C2C2-YABBY	3	1	PLATZ	1	4
C2H2	14	19	RWP-RK	4	2
C3H	16	24	S1Fa-like	1	1
CAMTA	0	3	SBP	3	5
CCAAT	9	11	Sigma 70-like	4	0
CPP	1	4	SRS	1	0
CSD	0	1	TAZ	1	1
DBP	2	3	TCP	4	6
E2F/DP	2	1	Tify	6	2
EIL	1	1	Trihelix	3	9
FAR1	5	10	TUB	2	5
FHA	7	6	ULT	1	0
G2-like	9	5	VOZ	0	2
GeBP	0	3	WRKY	22	10
GRAS	8	7	zf-HD	5	3
**Members of other transcriptional regulator gene families**					
**Gene family**	**Upregulated**	**Downregulated**	**Gene family**	**Upregulated**	**Downregulated**
ARID	2	1	PHD	12	22
AUX/IAA	3	19	Pseudo ARR-B	2	1
Coactivator p15	0	1	RB	0	1
DDT	3	0	Rcd1-like	0	2
GNAT	12	8	SET	11	6
HMG	4	5	SNF2	11	9
Jumonji	4	5	SOH1	0	2
LUG	0	2	SWI/SNF-BAF60b	4	4
MBF1	0	1	SWI/SNF-SWI3	2	1
MED6	0	1	TRAF	7	11

**Table 2:** Summary of spliced genes and events under dark and light conditions detected by the SpliceGrapher and the IsoLasso workflows. IR: intron retention; ES: exon skipping; Alt5′: alternative 5′ splice site; Alt3’: alternative 3′ splice site. Color scales: red (large counts) to blue (lower counts).

Chromosome #	Treatment	SpliceGrapher Prediction					IsoLasso				
		Genes	IR	ES	Alt5’	Alt3’	Genes	IR	ES	Alt5’	Alt3’
**1**	**Dark**	924	897	214	163	97	939	925	214	181	99
	**Light**	1028	994	264	184	109	1048	1021	264	191	112
**2**	**Dark**	747	728	224	167	109	771	781	227	189	117
	**Light**	802	777	219	153	99	822	812	222	165	103
**3**	**Dark**	825	797	216	136	94	858	868	216	162	120
	**Light**	945	893	219	143	98	960	920	219	153	118
**4**	**Dark**	565	551	182	112	64	581	576	183	117	67
	**Light**	596	572	193	104	61	611	592	194	109	66
**5**	**Dark**	519	503	139	139	70	538	534	140	114	75
	**Light**	552	537	144	103	80	562	550	144	109	92
**6**	**Dark**	509	459	186	128	62	527	493	186	135	64
	**Light**	531	501	169	103	63	554	535	169	109	67
**7**	**Dark**	466	432	166	77	48	482	468	166	85	51
	**Light**	481	473	129	72	52	498	498	129	82	56
**8**	**Dark**	400	390	97	63	62	415	416	98	68	64
	**Light**	463	433	134	62	84	475	457	134	67	87
**9**	**Dark**	341	342	79	53	32	349	359	79	64	37
	**Light**	375	361	118	49	38	382	377	118	53	39
**10**	**Dark**	290	275	94	43	29	295	288	94	49	36
	**Light**	308	295	102	61	32	314	307	102	65	35
**11**	**Dark**	304	266	113	49	35	313	278	113	53	36
	**Light**	363	306	150	51	35	374	325	150	57	37
**12**	**Dark**	324	310	117	69	45	329	315	117	74	46
	**Light**	365	347	150	63	53	372	359	155	68	61

**Table 3:** Expression and splicing profile of genes in our data that are known to undergo splicing from previous reports.

Known Spliced Genes	Spliced in	Light Regulation	Gene Description
Os01g0619000	Dark, Light	Down	RNA recognition motif domain-containing protein
Os01g0649900	Dark, Light	-	GDSL esterase/lipase protein 20
Os01g0764000	Dark, Light	Up	PHI glutathione s-transferase 2
Os01g0834400	Dark	-	HAP3A subunit of CCAAT-box binding complex
Os02g0122800	Dark, Light	Down	Similar to Arginine/serine-rich splicing factor
Os02g0130600	Dark	Up	Conserved hypothetical protein
Os02g0161900	Dark, Light	Down	Rice ubiquitin2
Os02g0197900	Dark, Light	Down	OsSTA53
Os02g0274900	Dark, Light	Up	Similar to metabolite transport protein CSBC
Os02g0577100	Dark, Light	Down	Zinc finger, RING/PHD-type domain containing protein
Os02g0666200	Light	Down	Aquaporin
Os02g0834000	Dark, Light	Up	Rac-like GTP-binding protein 5
Os02g0291000	-	Down	Calcineurin B-like protein 7
Os02g0291400	-	-	Calcineurin B-like protein 8
Os02g0823100	-	Up	Plasma membrane intrinsic protein 1;3
Os03g0265600	Dark, Light	Down	Transformer-2-like protein
Os03g0314100	Dark, Light	Down	DEAD-like helicase
Os03g0395900	Dark, Light	Down	Splicing factor
Os03g0670700	Dark, Light	Down	Glycine-rich RNA-binding protein 3
Os03g0698500	Dark, Light	Down	Similar to Yippee-like protein 3
Os03g0745000	Dark, Light	-	Heat stress transcription factor A-2a
Os03g0717600	-	Down	Zinc finger, C2H2-type
Os04g0115400	Dark, Light	Down	D111/G-patch domain containing protein
Os04g0649100	Dark, Light	-	Shattering abortion 1
Os04g0656100	Light	Down	H^+^-ATPase
Os04g0665800	Dark, Light	Up	Similar to H1005F08.12 protein
Os04g0402300	-	-	Cysteine-type peptidase
Os04g0479200	-	Up	Similar to NAD-dependent isocitrate dehydrogenase c;1
Os05g0348100	Dark	Up	Similar to CRR23 (chlororespiratory reduction 23)
Os05g0463800	Dark, Light	Down	HAP3C subunit of CCAAT-box binding complex
Os05g0554400	Dark, Light	Down	Phosphatidyl serine synthase family protein
Os05g0574700	Dark, Light	-	Similar to cDNA clone:002-182-C01
Os05g0534400	-	Up	Calcineurin B-like protein 4
Os05g0548900	-	Up	Phosphoethanolamine N-Methyltransferase 2
Os06g0172800	Light	-	Similar to alkaline alpha galactosidase 2
Os06g0651600	Dark, Light	Down	Protein phosphatase 2C58
Os06g0727200	Dark	Down	Catalase B
Os06g0128500	-	Up	Ribosomal protein L47, mitochondrial family protein
Os06g0133000	-	-	Glutinous endosperm, waxy
Os06g0506600	-	-	Ubiquitin-conjugating enzyme 17
Os07g0490400	Dark, Light	Up	FK506 binding protein 20-2
Os07g0574800	-	Up	Tubulin alpha-1 chain
Os07g0613300	-	-	Similar to PAUSED
Os08g0191600	Dark, Light	Up	Autophagy associated gene 8C
Os08g0530400	Dark, Light	Up	Moco containing protein, similar to sulfite oxidase
Os08g0436200	-	-	Zinc finger, RING/PHD-type domain containing protein
Os10g0115600	Dark, Light	Down	U1 snRNP 70K
Os10g0535800	Dark, Light	-	Cys-rich domain containing protein
Os10g0564900	Dark	Down	Similar to protein kinase CK2 regulatory subunit CK2B2
Os10g0567400	Dark, Light	Up	Chlorophyll a oxygenase 1
Os10g0577900	Dark, Light	-	Glycerol-3-phosphate acyltransferase
Os10g0411500	-	Down	Q calmodulin-binding region domain containing protein
Os11g0157100	Dark, Light	Down	Cyclin-T1-4
Os11g0700500	Dark	-	MybAS1
Os11g0600700	-	Up	Zinc finger, RING domain protein
Os12g0567300	Dark	Up	MybAS2, R2R3-Myb
Os12g0632000	Dark, Light	Down	Glycine-rich Protein GRP162

## Data Availability

The sequence reads and metadata are accessible from EMBL-EBI ArrayExpress (accession number E-MTAB-5689). The analyzed dataset is available at Gupta and Jaiswal, 2024 [112].

## Supplementary Materials

The following supporting information can be downloaded at: https://www.mdpi.com/article/10.3390/genes15081072/s1, Figure S1: Expression pattern of differentially expressed genes (14,766) with target FDR controlled at 5%. Figure S2: Gene Ontology enrichment analysis of differentially expressed genes. Figure S3: Bar plots of most significantly enriched GO terms for DES genes with -log10-transformed FDR values. Light-upregulated (green), light-downregulated (grey). Figure S4: Splicing events and expression pattern of circadian clock genes under dark and light conditions. Figure S5: Transcript graphs showing splicing of *Casein kinase beta subunit:CK2β-1* (Os07g0495100), *Gigantea:GI* (Os01g0182600) genes during photomorphogenesis. Table S1: Summary of the RNA-Seq read mapping to the reference rice genome. Table S2: Differentially expressed genes. Table S3: Plant Reactome Pathway enrichment analysis for light-regulated genes. Table S4: Transcription factor genes regulated in light. Table S5: Transcription factors regulating MVA pathway genes. Table S6: lncRNA annotation using BLAST.
