# Correction: Gupta, P.; Jaiswal, P. Transcriptional Modulation During Photomorphogenesis in Rice Seedlings. *Genes* 2024, *15*, 1072

**DOI:** 10.3390/genes15121526

**Published:** 2024-11-27

**Authors:** Parul Gupta, Pankaj Jaiswal

**Affiliations:** Department of Botany and Plant Pathology, Oregon State University, Corvallis, OR 97331, USA; parul.gupta@oregonstate.edu

In the original publication [1], there was a mistake in the legend for Figure 6. The legend missed labeling in the splice graph of OS08G0468100 and mislabeled the splice graphs of OS08G0468700 and OS05G0503300. The correct legend appears below. Meanwhile, the correct title of Figure 6 should be “Figure 6: Splicing and expression pattern of nitrogen assimilation cycle genes under dark and light conditions. NRT: Nitrate transporter; NR: Nitrate reductase; NiR/SiR: Nitrite reductase/Sulfite reductase”. The authors state that the scientific conclusions are unaffected. This correction was approved by the Academic Editor. The original publication has also been updated.


