# Supplementary material for: Transcriptional Modulation during Photomorphogenesis in Rice Seedlings
Source: Genes (Basel). 2024 Aug 14;15(8):1072. doi: 10.3390/genes15081072 (PMC11353317; doi:10.3390/genes15081072)
Supplement: Supplementary file 1 [file genes-15-01072-s001.zip › Supplementary Figure S3.pdf]

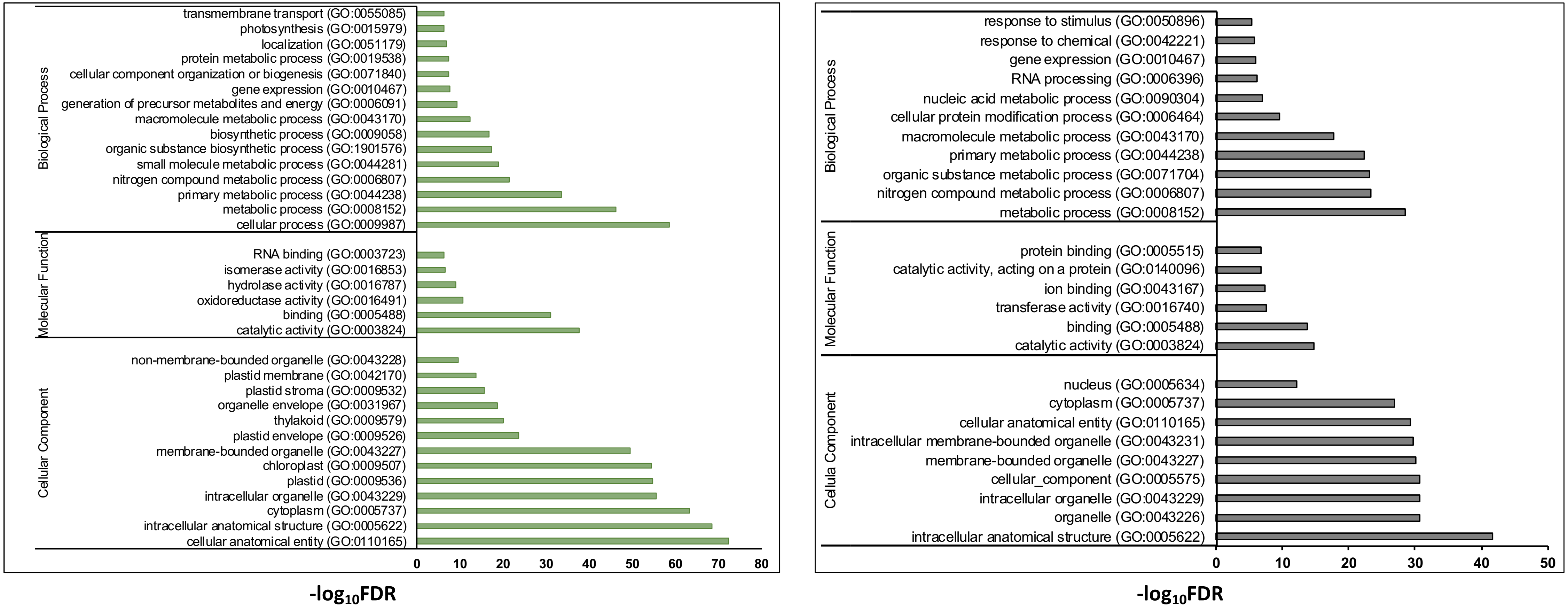

Supplementary Figure S3: Bar plots of most significantly enriched GO terms for DES genes with $-\log_{10}$ transformed FDR values. Light-upregulated (green), light-downregulated (grey).
