# Supplementary material for: Transcriptional Modulation during Photomorphogenesis in Rice Seedlings
Source: Genes (Basel). 2024 Aug 14;15(8):1072. doi: 10.3390/genes15081072 (PMC11353317; doi:10.3390/genes15081072)
Supplement: Supplementary file 1 [file genes-15-01072-s001.zip › Supplementary Figure S4.pdf]

Genes	Light				Dark			
	IR	ES	Alt5'	Alt3'	IR	ES	Alt5'	Alt3'
CK2α-1 (Os03g0762000)	-	-	-	-	1	-	-	-
CK2α-2 (Os03g0763000)	1	1	1	-	1	-	-	-
CK2β-2 (Os10g0564900)	-	-	-	-	1	-	-	-
PhyB (Os03g0309200)	-	-	-	-	1	-	-	-
TOC1 (Os02g0618200)	-	-	-	-	2	-	-	-
PIF3 (Os01g0286100)	1	-	-	-	-	-	-	-
CRY2 (Os02g0625000)	2	-	-	-	1	-	-	1
PRR37 (Os07g0695100)	3	1	-	1	2	-	-	-
PRR73 (Os03g0284100)	3	-	2	-	2	1	-	3
PRR95 (Os09g0532400)	2	-	1	-	1	-	1	-
LHY/CCA1 (Os08g0157600)	-	1	-	-	1	-	-	-

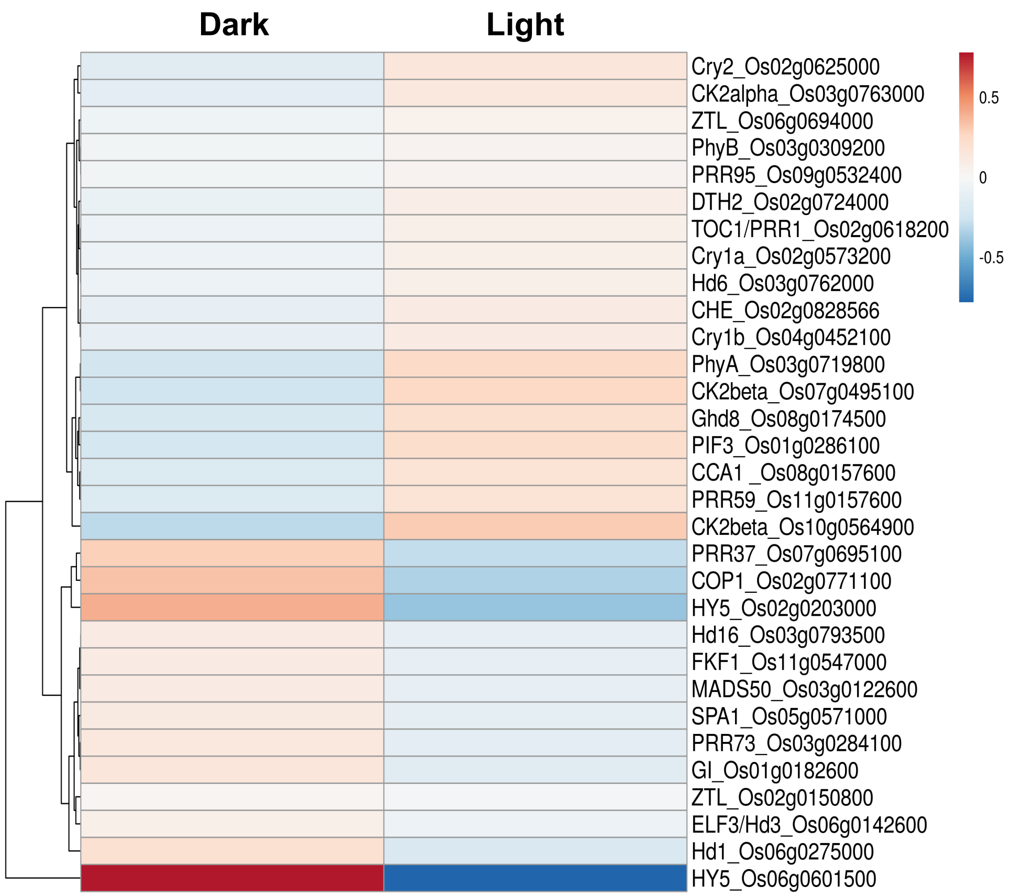

Supplementary Figure S4: Splicing events and expression pattern of circadian clock genes under dark and light conditions.
