# Supplementary material for: Transcriptional Modulation during Photomorphogenesis in Rice Seedlings
Source: Genes (Basel). 2024 Aug 14;15(8):1072. doi: 10.3390/genes15081072 (PMC11353317; doi:10.3390/genes15081072)
Supplement: Supplementary file 1 [file genes-15-01072-s001.zip › Supplementary Table S1.pdf]

Supplementary Table 1: Read mapping summary of rice seedlings grown in continuous dark and exposed to light.

Sample	Input reads	High quality reads	Reads mapped to genome
Dark-Rep1	13,760,553	13,001,563	12,249,629
Dark-Rep2	14,147,179	13,335,860	12,324,418
Dark-Rep3	13,442,449	12,505,262	11,783,274
Light-Rep1	14,554,961	13,625,166	12,677,035
Light-Rep2	14,577,314	13,755,841	13,040,444
Light-Rep3	13,333,010	12,695,151	12,058,127
